# An Institutional Survey of Early-Onset Breast Cancer in Young Women With Imaging and Molecular Subtype Correlation

**DOI:** 10.7759/cureus.91518

**Published:** 2025-09-03

**Authors:** Harini Gnanavel, Bhawna Dev, Jai Prakash Srinivasan, Archana Balasubramanian

**Affiliations:** 1 Department of Radiology, Sri Ramachandra Institute of Higher Education and Research, Chennai, IND; 2 Department of Pathology, Sri Ramachandra Institute of Higher Education and Research, Chennai, IND

**Keywords:** breast cancer, high-risk screening, imaging, molecular subtype, young women

## Abstract

Purpose

The burden of breast cancer in young women is high, necessitating an effective breast imaging screening protocol to decrease morbidity and mortality. This is one of the few studies conducted in India on young women with breast cancer, where we present imaging features along with proven molecular subtyping.

Methods

This is a retrospective study of 58 young women with biopsy-proven breast cancer between the period of January 2021 to December 2023. All imaging features were analyzed, and histopathological correlation was done.

Results

In our cohort, the most common age group was between 35 and 40 years. All cases underwent ultrasound, while X-ray and mammography were performed in 20 cases. Among these, three patients underwent contrast-enhanced digital mammography (CEDM), and two underwent MRI. The majority of malignancies were diagnosed as invasive mammary carcinoma. Regarding molecular subtypes, Luminal B was the most prevalent, which was a unique finding in our study, followed by triple-negative breast cancer (TNBC). Lesions with human epidermal growth factor receptor 2 (HER2)-neu and Luminal B subtypes predominantly exhibited indistinct margins, while TNBC cases commonly showed microlobulated margins. Malignant calcifications were most frequently seen in TNBC, followed by Luminal B. A one-way ANOVA demonstrated a statistically significant difference in Ki-67 levels among the subtypes. Tukey’s honestly significant difference (HSD) test for multiple comparisons revealed a significant difference between Luminal B and TNBC (p < 0.05), with TNBC showing a higher Ki-67 index. The chi-square test showed a statistically significant association between molecular subtype and the presence of metastases (p < 0.05), with Luminal B more commonly associated with metastatic disease. Pregnancy-associated breast cancer (PABC) was identified in four cases, of which two had metastases, suggesting that delayed presentation is common in PABC. Multicentric malignancies were relatively more frequent in the HER2-neu subtype.

Conclusion

Our study reflects the increase in breast cancer and metastases in young women. The Luminal B subtype was common in our study, which was different from other studies. Metastases and PABC were higher in Luminal B. Multicentric malignancy was relatively higher in HER2-neu. However, larger multicenter studies are recommended for validating this. These observations mandate an early imaging workup to detect cancer for better survival. We also recommend screening protocols as early as 35 years, as most of the presentations were above this age in our study.

## Introduction

Breast cancer is the fourth leading cause of cancer-related mortality worldwide, accounting for 11.6% of global cancer incidence in 2022, surpassing cervical cancer in prevalence [1-3]. Among women, breast cancer represents one in four cancer cases and is responsible for one in six cancer-related deaths [1-3].

The rising global trends in aggressive breast cancers among young women underscore the need for well-structured imaging screening protocols to facilitate early detection of early-onset breast cancer [3,4]. The implementation of advanced imaging technologies and adherence to established scientific guidelines are critical factors for early diagnosis and timely intervention [5].

Imaging strategies for young women with breast cancer should be personalized and stratified by age, particularly because no standardized screening guidelines exist for women under 40 years of age, except for those classified as high-risk. Although the imaging modalities remain consistent for younger women, mammography is often less effective due to the presence of dense breast tissue. In cases where mammography is required, digital breast tomosynthesis (DBT) is the preferred technique. MRI is recommended for high-risk screening or for evaluating disease extent following a breast cancer diagnosis in young women [6].

A significant challenge in this demographic is that young women often present with symptoms at an advanced stage of disease progression, rather than in the early stages. In light of this, we conducted a retrospective institutional survey and reviewed our experience with early-onset breast cancers. This study represents one of the few investigations in India focusing on breast cancer in young women, integrating imaging characteristics with confirmed molecular subtyping to enhance the understanding of this aggressive disease phenotype.

## Materials and methods

Patients

This study was approved by the ethics committee of Sri Ramachandra Institute of Higher Education and Research (approval IEC-NI/25/06/105/106), and informed consent was taken from all the patients enrolled in the study. In this retrospective study, a total of 2,223 consecutive young women under 40 years who came for breast imaging between the period of January 2021 and December 2023 were retrospectively reviewed in our Picture Archiving and Communication System (PACS) system. A total of 87 early-onset breast cancer patients were found, of whom the final cohort comprised 58 patients below the age of 40 years. Patients with benign breast mass (Breast Imaging Reporting and Data System (BI-RADS) 0, 1, 2, 3) and suspicious imaging findings who did not undergo biopsy in our institution and did not have immunohistochemistry (IHC) were excluded. 

Radiological evaluation and interpretation

Ultrasound images were acquired using a Toshiba Aplio 500 (Toshiba, Japan, 7-15 MHz linear probe) and a Samsung V8 (Samsung Healthcare, Seoul, South Korea, 2-14 and 4-18 MHz linear probes). All cases had ultrasound imaging done, followed by an X-ray mammogram for 20 cases, contrast-enhanced digital mammography (CEDM) for three cases, and two cases underwent MRI depending on the requirement of that particular case.

Mammograms were obtained in the standard craniocaudal (CC) and mediolateral oblique (MLO) views using a Fujifilm Amulet Innovality (Fujifilm Healthcare, Lexington, US) unit. In our institution, mammography is not routinely performed in women under 40 years of age, and ultrasound is the initial imaging modality in patients less than 40 years of age. If a sonographically suspicious finding is detected or in high-risk cases, a mammogram is done from 30 years on in our institution, followed by contrast MRI or CEDM for the detection of additional malignant foci wherever required.

The MRI scans were acquired with the patient in the prone position in a GE Architect (3T MRI, GE Healthcare, Boston, US) unit equipped with a breast coil. The MR images were acquired using the following sequences: an axial, turbo spin echo T2-weighted imaging sequence and a pre- and post-contrast axial T1-weighted flash three-dimensional volumetric interpolated breath-hold examination (VIBE) sequence obtained before and at 7, 67, 127, 187, 247, and 367 s after a bolus injection of 0.1 mmol/kg Gd-DTPA.

CEDM images were acquired using a Fujifilm Amulet Innovality (Fujifilm Healthcare, Lexington, US) unit. The contrast agent used was Visipaque in a dosage of 1.5 ml/kg body weight, and recombined images were interpreted.

All mammography, US, and MRI findings were retrospectively interpreted by two certified radiologists with eight and 20 years of experience, without knowledge of the image findings of other modalities. The nomenclature used was the morphological criteria described for mammography, US, and MRI in the American College of Radiology (ACR), BI-RADS lexicon, fifth edition [7].

Biopsy and pathological analysis

All BI-RADS 4, 5, and 6 lesions were subjected to image-guided Tru-Cut biopsy followed by an axillary lymph node fine needle aspiration cytology (FNAC) in cases of suspicious nodes. When a patient had multiple target lesions in the same or contralateral breast, all the lesions were biopsied.

Histopathologic reports were reviewed from the archives of the Department of Pathology. The following information was retrieved and reviewed: tumor location, focality, histologic type, tumor size, Nottingham’s histologic grade based on tubular/glandular differentiation, nuclear pleomorphism and mitotic rate, presence of lymphovascular or perineural invasion, axillary lymph node metastasis present or absent, and treatment effect (Residual Cancer Burden) [8]. Further, molecular subcategorization based on IHC and expression of markers was assessed and classified into Luminal A, Luminal B (Ki67>14% or PR>20%), human epidermal growth factor receptor 2 (HER2)-neu (Luminal HER2-neu or enriched), and triple-negative (basal-like or unclassifiable) categories. IHC analysis was performed by two independent pathologists to avoid bias. Estrogen receptor (ER) and progesterone receptor (PR) status were assessed based on the Allred scoring system. For cases that fell into the HER2/neu equivocal category, a HER2/neu by fluorescence in situ hybridization (FISH) was performed and reclassified accordingly.

Statistical analysis

The data were processed using SAS version 9.2 (SAS Inc., Cary, NC, US). A one-way ANOVA and Tukey's honestly significant difference (HSD) test were used to examine differences in continuous variables, and the chi-square test was used for categorical variables. Significance was assumed for p-values < 0.05.

## Results

Patient demographics

In our analysis of young women less than 40 years old, 87 cases of breast malignancy in young women were found, out of which only 58 cases were included in the study. The cases that did not have IHC, imaging workup, or histopathological examination (HPE) done outside our institution were not included. The common age group fell between 35 and 40 years, with a lump being the predominant presenting complaint. 

Imaging

**Figure 1:**
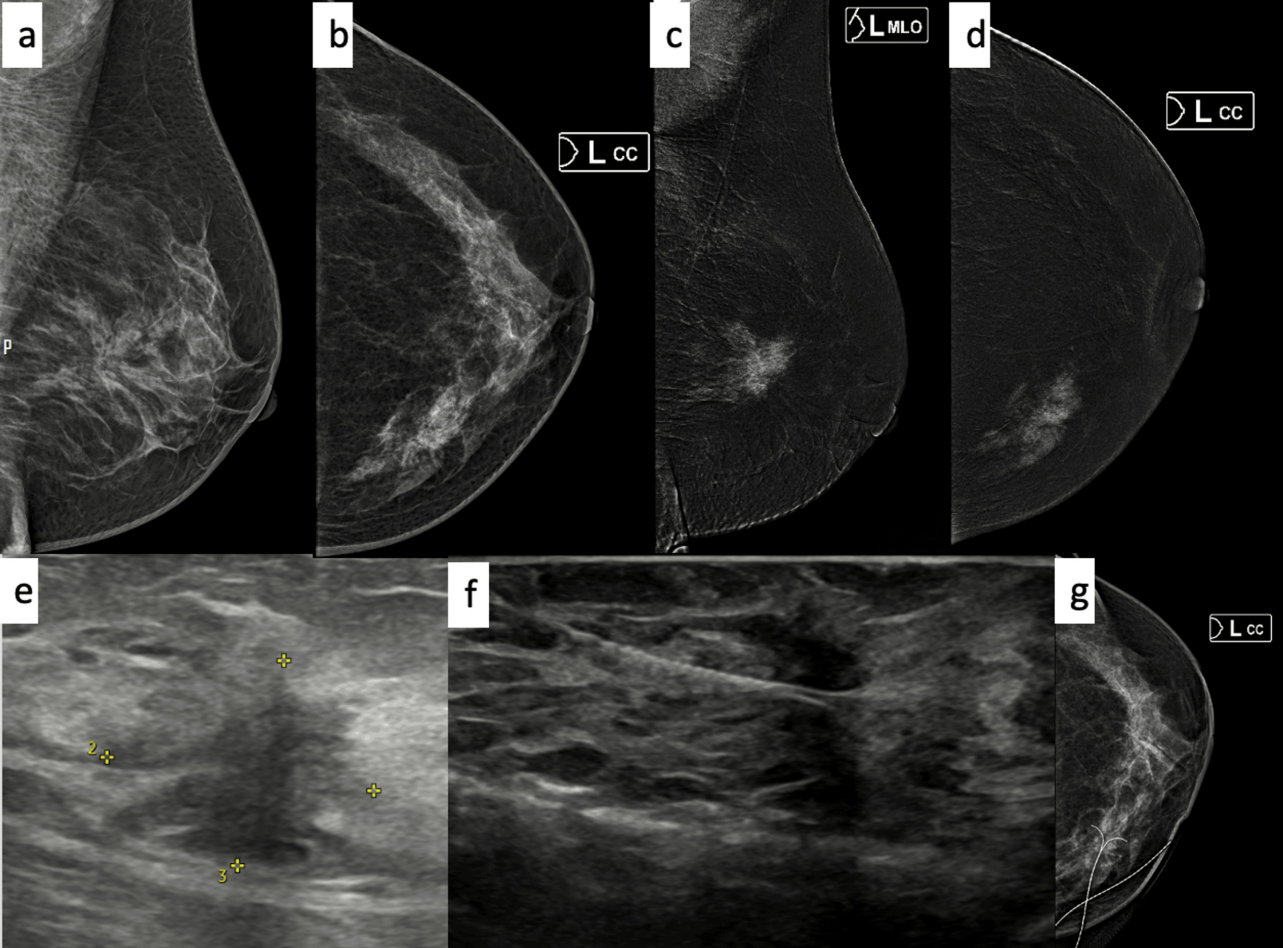
A case of a 32-year-old female a,b: X-ray mammogram MLO and CC views of the left breast show an architectural distortion (AD) with an underlying irregular mass in the inner central quadrant; c,d: CEDM recombined images show heterogeneous enhancement in the region of AD and mass; e: ultrasound shows an irregular taller-than-wide spiculated mass with surrounding AD; f,g: ultrasound-guided wire localization with a check X-ray mammogram. CC: craniocaudal; MLO: mediolateral oblique; CEDM: contrast-enhanced digital mammography

**Figure 2:**
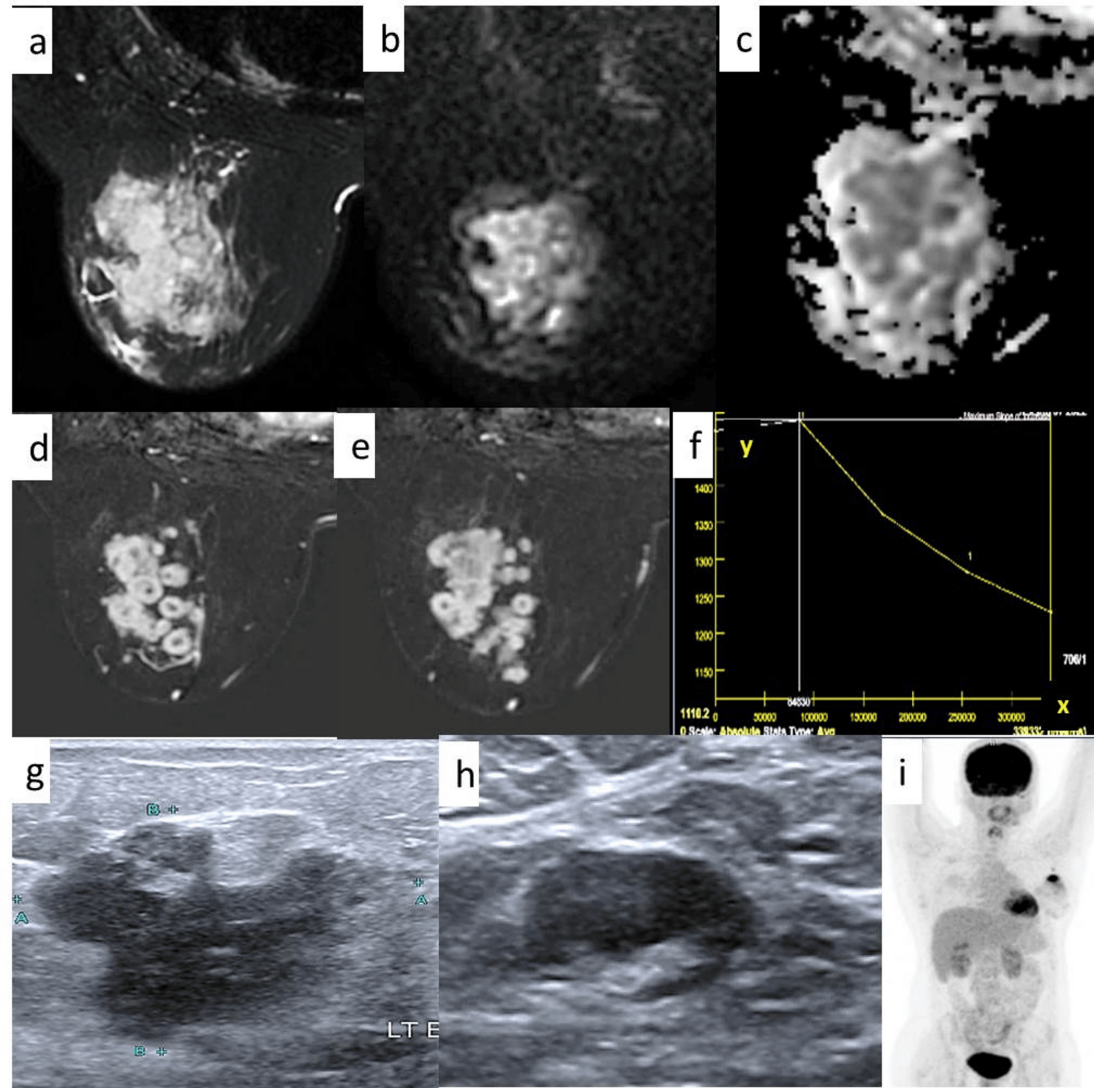
A case of a 23-year-old female a-c: T2-weighted, DWI, and ADC images show a T2 isointense non-mass area with diffusion restriction and a corresponding drop in signal in ADC; d,e: MRI dynamic contrast images show non-mass clumped ring enhancement in diffuse distribution; f: MRI shows a time intensity curve with a type 3 kinetic curve, with the x and y axis representing the time and signal intensity, respectively; g,h: ultrasound shows an irregular hypoechoic mass and a lymph node with cortical thickening in the ipsilateral axilla; i: FDG PET scout image shows no distant metastases. DWI: diffusion weighted imaging; ADC: apparent diffusion coefficient; FDG: fluorodeoxyglucose

**Figure 3:**
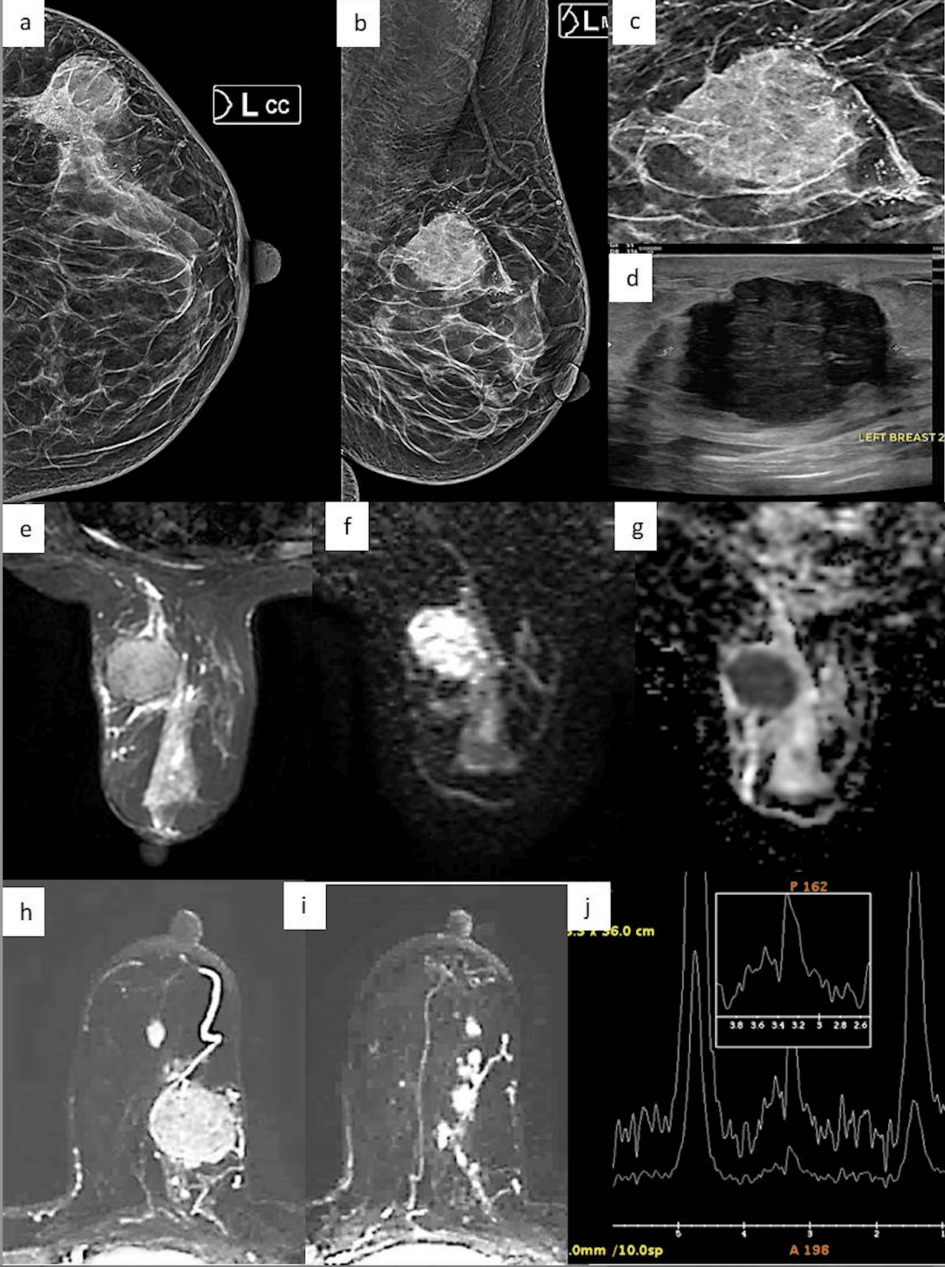
A case of a 35-year-old female a,b: X-ray mammogram CC and MLO views of the left breast show a round circumscribed mass with microcalcifications in the upper outer quadrant; c: zoomed images of the X-ray mammogram show the mass with microcalcification; d: ultrasound shows an irregular hypoechoic microlobulated mass with posterior enhancement; e: MRI T2-weighted image show a T2 isointense microlobulated mass; f,g: DWI and ADC images show a mass with diffusion restriction and a drop in ADC signals within the mass; h,i: post-contrast MIP images show an enhancing mass with adjacent non-mass enhancement in the linear distribution corresponding to the area of microcalcifications on mammogram; j: MR spectroscopy shows a choline peak at 3.2 ppm represented as a graph with x and y axis showing the degree of chemical shift expressed in part-per-million (ppm). CC: craniocaudal; MLO: mediolateral oblique; MIP: maximum intensity projection; DWI: diffusion weighted imaging; ADC: apparent diffusion coefficient

Ultrasound was done for all cases, and X-ray mammography was done for 20 cases, of which three underwent CEDM (Figure 1), and two cases underwent MRI (Figures 2, 3).

On ultrasound, the imaging features of the lesions were predominantly irregular shape (n=47, 81%) and indistinct margins (n=26, 44.8%) (Table 1). X-ray mammograms of the 20 cases showed the following margins: indistinct n=11 (18.9%), partially obscured n=3 (5.1%), spiculated n=4 (6.9%), and not visualized due to dense breast n=2 (3.4%).

**Table 1:** Correlation between ultrasound characteristics, pathological and molecular behaviour in young women with breast cancer TNBC: triple-negative breast cancer; HER2: human epidermal growth factor receptor 2

Characteristics		Number %	Luminal A&B	HER2-neu	TNBC
Shape	Round	9 (15.5%)	3 (12.50%)	1 (6.25%)	5 (27.78%)
	Oval	2 (3.4%)	Nil	2 (12.50%)	Nil
	Irregular	47 (81%)	21 (87.5)	13 (81.25%)	13 (72.2)
			24	16	18
Margins	Circumscribed	5 (8.6%)	1 (4.17%)	3 (18.75%)	1 (5.56%)
	Indistinct	26 (44.8%)	13 (54.5%)	7 (43.75%)	6 (33.2%)
	Angular	9 (15.5%)	5 (20.83%)	3 (18.75%)	1 (5.56%)
	Microlobulated	16 (6%)	4 (16.67%)	2 (12.50%)	10 (55.56%)
	Spiculated	2 (3.4%)	1 (4.17%)	1 (6.25%)	Nil
			24	16	18
Calcification	Present	13	6 (46.15)	5 (38.46)	2 (15.3)
Posterior features	Combined	17	8 (33.2%)	7 (43.7%)	2 (11.6%)
	Shadowing	13	6 (25%)	3 (18.7%)	4 (23.5%)
	Enhancement	13	3 (12.5%)	4 (25%)	6 (35.2%)
			17	14	12
Lymph node	Yes	27	10 (37.0%)	8 (29.6%)	9 (33.3%)

Correlation of radio-pathological data

**Figure 4:**
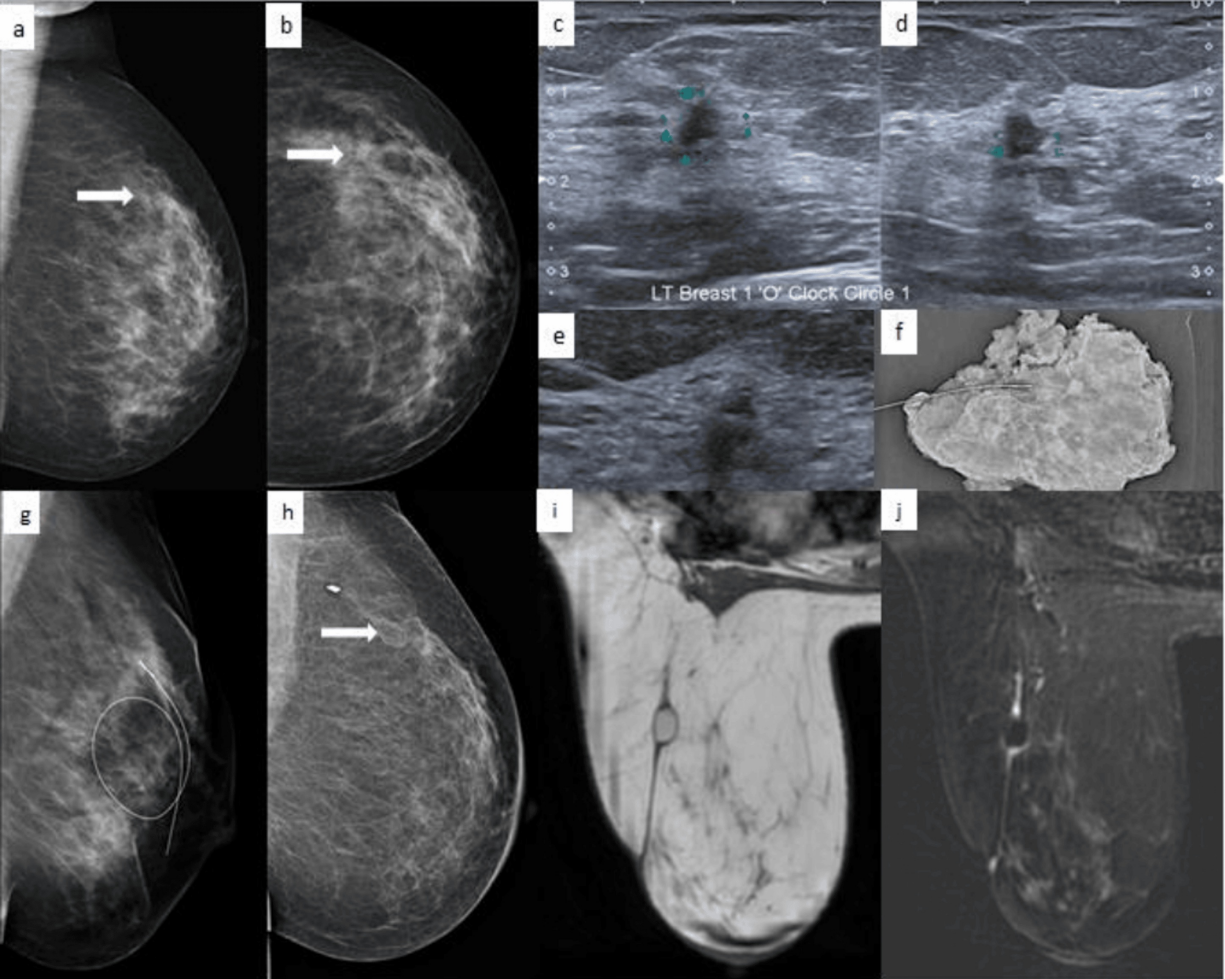
A case of a 36-year-old female a,b: X-ray mammogram MLO and CC views of the left breast show subtle architectural distortion in the upper outer quadrant (arrow); c,d: ultrasound shows an irregular hypoechoic lesion, taller than wider lesion in transverse and longitudinal sections; e-g: wire localization on ultrasound, specimen mammogram, check X-ray mammogram with wire in situ; h: follow-up X-ray mammogram after six months shows an area of fat necrosis (arrow) with a clip in situ; i,j: MRI post one year, T1 and T1 FS images show the same area of fat necrosis showing suppression of fat. CC: craniocaudal; MLO: mediolateral oblique; T1 FS: T1-weighted fat-suppressed

The majority of malignancies were invasive mammary carcinoma, n=40 (69%). In the molecular subtype, Luminal B represented the majority (n=22, 37.9%), followed by TNBC (n=18, 31%), HER2-neu (n=16, 27.6%), and Luminal A (n=2, 3.5%). The mean age was 36 years for Luminal B, 34 years for triple-negative breast cancer (TNBC), and 37 years for HER2 neu (Table 2). On ultrasound, HER2-neu (n=7, 43.8%) and Luminal A and B combined (n=13, 54.5%) had indistinct margins predominantly, and TNBC had microlobulated margins (n=10, 55.6%) (Figure 3). Calcification was seen in 13 cases (22.4%); Luminal B had six cases of calcification, HER2-neu had five, and TNBC had two cases (11.1%) (Table 1). However, when correlated with the percentage of TNBC cases presenting with malignant calcification to Luminal B cases presenting as calcification, TNBC had a higher percentage of malignant calcification, followed by Luminal B. Associated features that were seen were nipple retraction (n=2, 3.4%) and architectural distortion (n=5, 6.9+1.7%) (Figure 4).

**Table 2:** Frequency analysis of breast malignancy in young women regarding age and pathological behavior TNBC: triple-negative breast cancer; HER2: human epidermal growth factor receptor 2

Age	Number%	Luminal B	Luminal A	HER2-neu	TNBC
20-25	1 (1.7%)	0 (0)	0 (0%)	0 (0 %)	1 (5.6 %)
26-30	3 (5.2%)	1 (4.6 %)	0 (0%)	1 (6.25 %)	1 (5.6 %)
31-35	12 (20.7%)	5 (22.7 %)	0 (0%)	2 (12.5 %)	5 (27.8 %)
36-40	42 (72.4%)	16 (72.7 %)	2 (100%)	13 (81.25 %)	11 (61.2%)

The mean Ki-67 for the cases was Luminal B - 48.7% (18.97 SD), TNBC - 65% (19.2 SD), and HER2-neu - 54.8% (17.16 SD) (Table 3). A one-way ANOVA was performed to compare the Ki-67 values with different molecular subtypes, which revealed that there was a statistically significant difference in Ki-67 between groups (F(2, 53)=4.08, p = 0.02). Tukey’s HSD Test for multiple comparisons found that there was a statistically significant difference between Luminal B and TNBC (p < 0.017), with TNBC having higher Ki67. Two cases of Luminal A were not included since they did not contribute much to the statistical value. 

**Table 3:** Frequency analysis of Ki-67 percentage, mean age according to molecular subtype in young women IHC: immunohistochemistry; TNBC: triple-negative breast cancer

IHC	Number %	Ki-67% (mean)	Mean age
Luminal B	22 (37.9 %)	48.7	36
HER2-neu	16 (27.6%)	54.8	37
TNBC	18 (31.0 %)	65	34
Luminal A	2 (3.5 %)	45	36

Spread to the ipsilateral axillary lymph node was seen in n=27 (46.6%) cases, with Luminal B (10 cases) having higher positivity. Metastases were seen in 12 cases (n=12, 20.7%) with a predominance in Luminal B (eight cases). The chi-square test showed statistical significance between the molecular subtype and metastases (p<0.05), with Luminal B having metastases more commonly. 

Multicentric malignancy was identified in seven cases, of which three were HER2-neu, two were TNBC, and two were Luminal B.

Pregnancy-associated breast cancer

**Figure 5:**
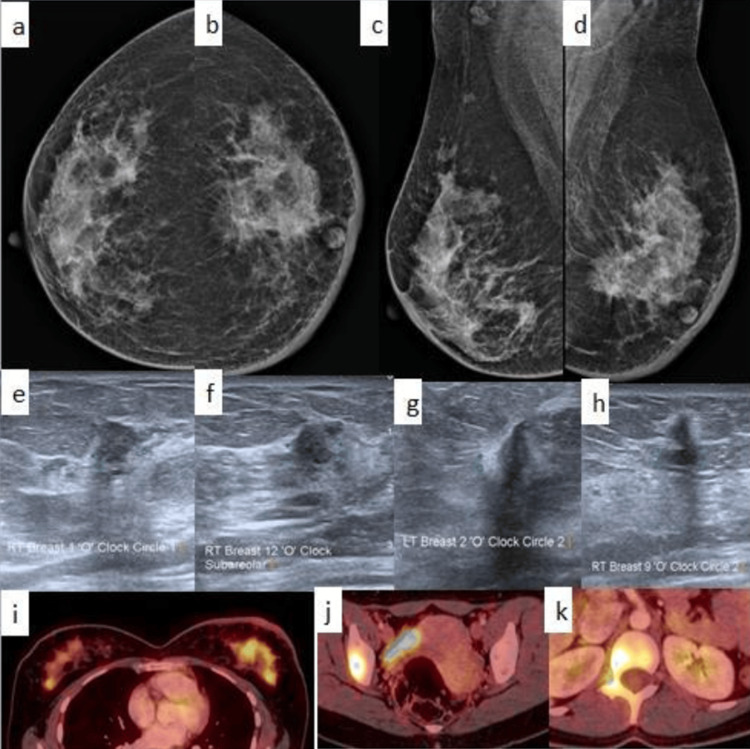
A case of a 29-year-old lactating female with pregnancy-associated breast cancer a-d: bilateral CC and MLO views on X-ray mammogram show lesions in both breasts with partially obscured margins and surrounding architectural distortion; e-h: ultrasound shows multiple bilateral irregular lesions with angular margins; i-k: FDG PET shows bony metastases with FDG-avid lesions in both breasts, the right iliac bone, and the vertebra. CC: craniocaudal; MLO: mediolateral oblique; FDG: fluorodeoxyglucose

Pregnancy-associated breast cancer (PABC) was present in four cases, of which two cases had metastases (Figure 5), implying the factor that presentation is late in PABC. Of these, three cases were Luminal B subtype.

## Discussion

Breast cancer is the second most frequently diagnosed cancer worldwide and the fourth leading cause of cancer-related mortality, accounting for 6.9% of cancer deaths and 11.6% of the global cancer burden [2]. Young women have a higher incidence of tumors with poor prognostic features, including negative estrogen receptor (ER−) status, larger tumor size (> 2.0 cm), HER2 overexpression, epidermal growth factor receptor expression, lymph node involvement, and high nuclear grade. However, despite these aggressive characteristics, estrogen receptor-positive (ER+) Luminal tumors remain the most prevalent subtype in young women [9]. Among the Luminal subtypes, Luminal B is the most common due to its frequent co-expression of HER2 and/or high proliferative activity. Studies have shown that women under the age of 40 have a higher likelihood of distant metastases [9,10], a finding consistent with our study, where Luminal B was the most frequently observed molecular subtype and was also associated with a higher incidence of metastases to distant organs. Metastatic disease was observed in 12 cases (20.7%), with Luminal B (eight cases) accounting for the majority. A chi-square test demonstrated statistical significance (p < 0.05) between molecular subtype and metastases, with Luminal B being the most commonly associated subtype.

Previous studies have reported invasive mammary carcinoma as the most frequent histological type in young women, which aligns with our findings [11]. In a study, most patients had no family history of breast cancer (60.7%) and were symptomatic at diagnosis (75.6%). The most common histological type was invasive carcinoma of no special type (NST), observed in 73.8% of cases. Several studies have reported Luminal B as the predominant molecular subtype, similar to our findings [12,13]. However, in a study by Rashmi Sudhir on young women with breast cancer, TNBC was the most prevalent subtype, accounting for 45.7% of cases. Additionally, tumors with HER2 overexpression were significantly associated with the presence of microcalcifications (p=0.006), while TNBC lesions were more likely to exhibit circumscribed margins [14]. In our study, Luminal B was the most frequent subtype, followed by TNBC. TNBC lesions predominantly exhibited microlobulated margins, with higher Ki-67 values and a higher incidence of microcalcifications, whereas Luminal B tumors were characterized by indistinct margins and a greater likelihood of distant metastases.

Another study reported that 91.8% of young women presented with a palpable mass, and imaging demonstrated multifocal or multicentric disease in 40.9% of cases, with Luminal subtypes being the most common [3]. In our study, multicentric malignancy was identified in seven cases: three were HER2-positive, two were TNBC, and two were Luminal B. Similarly, another study found that the majority of cases were in the 36-40 years age group, consistent with our findings. Lymphovascular invasion was present in 48.8% of cases, and 39 patients (45.3%) had TNBC [15]. In our study, ipsilateral axillary lymph node involvement was observed in 46.6% of cases, with Luminal B having the highest incidence.

Need for high-risk screening in young women

The increasing burden of early-onset breast cancer necessitates targeted high-risk screening strategies for young women. High-risk individuals are those with a lifetime breast cancer risk exceeding 20%, including those with genetic mutations (BRCA1, BRCA2, CHEK2, and TP53), a strong family history of breast cancer, or a history of chest irradiation at a young age. The ACR recommends annual contrast-enhanced breast MRI (CE-MRI) for high-risk women aged 25-29 years and both annual mammography and CE-MRI from age 30 onwards. Women treated with childhood radiation therapy should begin breast screening at age 25 or eight years after the completion of therapy, whichever is later. Supplemental ultrasound screening may be considered in select cases [9]. However, routine screening for the general population under 40 remains undefined, leading to delayed diagnoses, as observed in our study. Given that the majority of cases in our cohort occurred in women aged 35-40, we recommend initiating mammographic screening at age 35 to facilitate earlier detection and intervention.

Prognostic implications and survival disparities

Survival disparities in breast cancer have been noted based on age and molecular subtype, with Luminal subtypes exhibiting a wider survival gap compared to TNBC or HER2-positive disease [6]. PABC, defined as breast cancer diagnosed during pregnancy, within the first postpartum year, or during lactation, accounts for approximately 3% of all breast cancer cases [9]. The trend of delayed childbearing has led to an increased risk of developing breast cancer during pregnancy. PABC is an independent risk factor for poor prognosis, as these tumors often exhibit aggressive subtypes, frequently being ER-negative, a phenomenon thought to be linked to the downregulation of estrogen receptors during pregnancy [6]. The diagnostic approach typically begins with ultrasound, followed by mammography if ultrasound findings are suspicious or if a biopsy confirms malignancy [16]. Interestingly, in our study, Luminal B was the predominant molecular subtype in three out of four PABC cases, differing from previous reports. Given the small sample size, these findings require further validation in larger studies. Notably, tumors in our PABC cohort were aggressive, with metastases observed in two out of four cases.

Genetic and molecular insights

A comprehensive gene expression profiling study analyzing 40 datasets comprising 4,467 breast cancer cases found that younger age at diagnosis was associated with a higher frequency of HER2-positive and TNBC subtypes compared to Luminal subtypes, with TNBC carrying the poorest prognosis [17]. Additionally, a study evaluating racial disparities in early breast cancer molecular subtypes across five racial groups found that Indian women had a higher prevalence of TNBC and HER2-overexpressing subtypes compared to other racial groups, where Luminal subtypes were more common [18].

The European Society for Medical Oncology (ESMO) guidelines provide key recommendations for the screening, diagnosis, risk assessment, and management of early breast cancer [19]. Beyond IHC for molecular classification, gene expression profiling, germline genetic testing, and genetic counseling are recommended. The assessment of tumor-infiltrating lymphocytes (TILs) and programmed death-ligand 1 (PD-L1) expression may provide additional prognostic insights, although they are not currently used to guide treatment decisions [19].

Study limitations

Despite the valuable insights offered by this study, several limitations must be acknowledged. This was a single-institution study with a relatively small and heterogeneous sample size. However, given the rarity of breast cancer in women under 40, our findings remain significant and provide valuable correlations between molecular subtypes and clinical outcomes. A few early-onset breast cancer cases were excluded due to the unavailability of IHC and histopathology reports from external institutions, which was another relative limitation. A larger, multicenter study would further validate our findings. Nonetheless, this study is impactful, as it represents a diverse Indian population and is based in a high-volume tertiary referral center, enhancing its relevance.

## Conclusions

Our study highlights the increasing burden of breast cancer in young women, with a rising incidence of metastatic disease at the time of diagnosis. In our cohort, the Luminal B subtype was the most prevalent, followed by TNBC, differing from other studies where TNBC was more commonly reported. Imaging analysis revealed that indistinct margins were frequently observed in Luminal B tumors, whereas TNBC lesions predominantly exhibited microlobulated margins.

Metastases and PABC were more frequently observed in patients with the Luminal B subtype, with indistinct lesion margins as a characteristic imaging feature. TNBC cases demonstrated a higher incidence of calcifications and elevated Ki-67 values, indicating greater proliferative activity. Additionally, multicentric malignancies were relatively more common in the HER2-enriched subtype. However, larger multicenter studies are warranted to validate these findings and provide a more comprehensive understanding of disease patterns in young women.

These findings emphasize the urgent need to enhance awareness, promote self-examination, and ensure timely imaging evaluations to facilitate early detection and improve survival outcomes in young women. We advocate for the initiation of screening protocols above the age of 35 to enable earlier diagnosis and intervention in this high-risk population, as the majority of cases in our study fell above this age group.
